# More welcome data on pulmonary hypertension in South Africa

**DOI:** 10.7196/AJTCCM.2021.v27i1.142

**Published:** 2021-03-09

**Authors:** Richard Raine

**Affiliations:** Respiratory Clinic, Department of Medicine, Faculty of Health Sciences, University of Cape Town, South Africa

## Editorial

This issue of the AJTCCM contains more welcome data on the problem of pulmonary hypertension (PH) in South Africa (SA). The paper in this issue by Dahim *et al*. ^[1]^ describes the experience of the Pulmonary Hypertension Clinic at Inkosi Albert Luthuli Central Hospital and makes interesting comparisons with the data from Groote Schuur Hospital published in 2018.^[2]^ Retrospective data on 91 patients over an 8-year period are presented. This cohort excluded patients with type 2 PH, who were managed by cardiologists.

The trends are similar to those seen in Cape Town, with HIVassociated PH, idiopathic pulmonary arterial hypertension and chronic thromboembolic PH (CTEPH) being the most common causes seen. The fact that more than 30% of the patients had HIVassociated PH probably reflects the prevalence of disease and referral pattern in the area, although a high prevalence compared with the rest of the world was also reported for Cape Town. This was commented upon by Goolam Mahomed^[3]^ in a previous editorial.

The investigation of this group included echocardiography in all, although right-heart catheterisation was not performed in any patients. Monotherapy with sildenafil was used in 66%, with some evidence of a drop in pulmonary artery systolic pressure and functional improvement.

Twelve patients (13%) had CTEPH compared with the 38% seen in Cape Town. It seems that no specific therapy could be offered to these patients although international guidelines suggest that pulmonary endarterectomy be offered to eligible patients as this is associated with a significant survival advantage.^[4]^ Targeted medical therapy with riociguat and balloon pulmonary angioplasty have been suggested for those not suitable for pulmonary endarterectomy surgery.

Investigation and management of PH remains a major challenge in SA, particularly in the public sector, where access to right-heart catheterisation, reactivity testing and a variety of agents for treatment remains very limited, despite the large body of evidence and recommendations for these interventions.^[5,6]^
